# Perspective: Water-Filtered Infrared-A-Radiation (wIRA) – Novel Treatment Options for Chlamydial Infections?

**DOI:** 10.3389/fmicb.2019.01053

**Published:** 2019-05-10

**Authors:** Jasmin Kuratli, Nicole Borel

**Affiliations:** Institute of Veterinary Pathology, University of Zurich, Zurich, Switzerland

**Keywords:** *Chlamydia trachomatis*, wIRA irradiation, wIRA/VIS, wound healing, wound infection, alternative treatment strategies

## Abstract

Water-filtered infrared-A-radiation (wIRA) is a promising therapeutic method, which is particularly used as supportive treatment for wound closure, and wound infection treatment and prevention. High penetration properties of the heat field and beneficial effects on wound healing processes predispose wIRA irradiation to be a non-invasive treatment method for bacterial infections in superficial tissues. Since *Chlamydia trachomatis* still represents the leading cause of infectious blindness in third world countries (WHO http://www.who.int/topics/trachoma/en/) and wIRA displays beneficial effects on chlamydial infections *in vitro* without inducing cellular damage in *ex vivo* eye models and also shows beneficial effects on wound healing, this irradiation technique might represent a promising future treatment for trachoma patients. To this end, further studies investigating shorter irradiation times or irradiation of *Chlamydia* in chronic infections [the chlamydial stress response (Bavoil, 2014)] as well as safety studies in animal models should clearly be performed.

## Water-Filtered Infrared-A-Radiation (wIRA)

Electromagnetic radiation with wavelengths between 760 nm (0.760 μm) and 1000’000 nm (1000 μm) is called infrared radiation (IR). This spectrum is divided into the three sub-ranges of IR-A (760 – 1400 nm), IR-B (1400 – 3000 nm), and IR-C (3000 – 1000’000 nm and 3.0 – 1000 μm) (Cobarg, 1995). The water-filtered IR-A-radiation (wIRA) shows a high degree of accordance with the terrestrial IR solar spectrum at noon as well as a strong reduction of emission within the water absorption bands at wavelengths of 840, 970, 1200, and 1470 nm with negligible contributions of IR at wavelengths longer than 1400 nm (Cobarg, 1995). The radiation of a halogen bulb is filtered through a water-filled cuvette in special irradiators (Cobarg, 1995; Rzeznik, 1995). wIRA attains high tissue penetration with low thermal loads to the skin surface (in contrast to other sources of IR without the above-mentioned properties) and increases tissue temperature, oxygen partial pressure and tissue perfusion (Hellige et al., 1995; Hartel et al., 2007; Hoffmann, 2007). The most important clinical effects of wIRA reported in patients include reduction of pain, inflammation, hypersecretion and improved wound healing (Hartel et al., 2006, 2007; Hoffmann, 2007; Mercer et al., 2008). wIRA in the clinical setting of wound healing has been extensively reviewed elsewhere (Hartel et al., 2007; Hoffmann, 2007, 2009; von Felbert et al., 2007; Hoffmann et al., 2016).

## Application of wIRA in Patients

A variety of clinical applications for wIRA has been described (reviewed in Hoffmann, 2007, 2017a), like improvement of wound healing in acute and chronic wounds (Hartel et al., 2007; von Felbert et al., 2007; Hoffmann, 2009; Daeschlein et al., 2012; Hoffmann et al., 2016), application in sports medicine and physiotherapy (Vaupel et al., 1995; Hoffmann, 2002), treatment of Morbus Bechterew (Falkenbach et al., 1996) or weight loss (Möckel et al., 2006), in the neonatology field (Singer et al., 2000; Löwe and Singer, 2008), for treatment of scleroderma lesions (Von Felbert et al., 2007, 2011), swallowing disorders and hypersalivation (Hoffmann, 2017b), therapy of recurrent breast cancer (Notter et al., 2017) and superficial tumors (Vaupel et al., 2018), or as a treatment method for actinic keratosis in combination with photodynamic therapy (PDT) (Von Felbert et al., 2010; Giehl et al., 2014). Moreover, wIRA can promote the penetration of topically applied substances (Otberg et al., 2008) and is also attractive for cosmetics and wellness applications (Piazena and Kelleher, 2010; Jung and Grune, 2012).

First introduced for chronic wound treatment in 1989, newer reports also recommend wIRA as a means to improve acute wound healing (Hartel et al., 2007; Daeschlein et al., 2012). After first anecdotal reports appeared describing single cases of better wound healing and reduced pain in patients following wIRA treatment, randomized control studies confirmed the beneficial effects of wIRA irradiation (reviewed in Hartel et al., 2007; von Felbert et al., 2007; Hoffmann, 2009; Hoffmann et al., 2016).

Re-irradiation of local recurrent breast cancer is often challenging and, in severely pre-treated patients, also limited due to risks of skin damage (Notter et al., 2017). A combination of superficial hyperthermia and radiation therapy allows reduction of the total radiation dose while achieving local control (Notter et al., 2017). wIRA irradiation has been assessed as a potential contactless heating technique, which is especially promising in the treatment of large superficial, neoplastic skin areas, and ulcerated or bleeding lesions due to additional wound healing promoting effects (Notter et al., 2017; Vaupel et al., 2018). First applications of water-filtered infrared-A and visible light irradiation (wIRA/VIS) in combination with PDT resulted in reduced local pain compared to PDT with light emitting diodes (LED) (Von Felbert et al., 2010) or with the light of a halogen bulb (Giehl et al., 2014). In addition, wIRA treatment improved PDT efficacy by inducing increased blood flow and tissue oxygenation (Von Felbert et al., 2010). wIRA can, moreover, be used in the treatment of other tumors such as malignant melanoma, vulvar carcinoma, skin metastases of different primary tumors, cutaneous T- and B-lymphomas, cutaneous large-area hemangiomatosis, inoperable squamous cell, basal cell and eccrine carcinoma (Vaupel et al., 2018).

Besides these myriad indications (reviewed by Hoffmann, 2007), wIRA application has been shown to be beneficial for the clinical treatment of donor site infections in split-thickness skin grafts (Aljasir et al., 2018). Hartel et al. (2006) postulated the tendency of reduced wound infection rates upon postoperative wIRA treatment. Preoperative wIRA application in a study with 400 patients undergoing abdominal surgery significantly reduced surgical site infections (Künzli et al., 2013). The combination of wIRA with PDT showed beneficial effects in the antimicrobial treatment of planktonic cultures and subgingival biofilms (Al-Ahmad et al., 2013, 2016; Karygianni et al., 2014). Furthermore, intracellular bacteria (*Chlamydia pecorum* and *Chlamydia trachomatis*) were significantly reduced *in vitro* by wIRA alone or in combination with visible light (wIRA/VIS) (Marti et al., 2014, 2015; Rahn et al., 2016; Kuratli et al., 2018).

## wIRA-Induced Clinical Observations

The most important and reproducible clinical observations on wIRA treatment (increased tissue temperature, oxygen partial pressure, and tissue perfusion with decreased pain) are summarized in the first section of this report.

Wound centers are often relatively or absolutely hypothermic compared to the margins of the wound or unchanged skin areas (Hoffmann, 2007; Mercer et al., 2008), resulting in impaired wound healing; however, this can be treated by wIRA-induced elevation of local tissue temperature (Hoffmann, 2007). Reduced local oxygen partial pressures, often present in chronic wounds, inhibit sufficient energy production for wound healing processes (e.g., respiratory burst of granulocytes) and predispose to wound infections (Hoffmann, 2007). wIRA treatment can increase oxygen partial pressure, which has been demonstrated by implanted probe measurements (Hartel et al., 2006). The accumulation of lactate (leading to acidosis) and inflammatory metabolites as a result of impaired blood circulation can induce pain. Patients reported less pain perception during and after wIRA treatment (Hartel et al., 2006, 2007). Improved microcirculation due to wIRA treatment leads to removal of pain metabolites and long-lasting increase in tissue temperature through improved blood flow (Hoffmann, 2007).

## Working Mechanism of wIRA

Physically, wIRA is a type of thermal radiation inducing thermal as well as non-thermal effects (Hoffmann, 2007). Thermal effects are explained by the increased tissue temperature and the increased kinetic energy (energy absorption) of water molecules upon heat radiation, leading to temperature-dependent changes in the affected tissue (Burri et al., 2004; Vaupel et al., 2018). Non-thermal effects are independent of temperature changes and a result of direct stimulation of cells or cellular structures (Burri et al., 2004). However, as stated by Jung et al. (2012), discriminating between thermal and non-thermal effects can be difficult when temperature changes are not strictly controlled. In cell culture, in contrast to skin models or even to a patient’s skin, there is only one cell monolayer and the protective epidermis or temperature control by blood circulation is non-existent (Jung et al., 2010, 2012).

On the cellular level, cytochrome c oxidase has been extensively discussed as a potential target of visible light and near-infrared treatment (Karu, 1999, 2010; Karu et al., 2005). Cytochrome c oxidase is a large multicomponent membrane protein and the terminal enzyme of the respiratory chain in eukaryotic cells which mediates the transfer of electrons from cytochrome c to molecular oxygen (Karu, 1999). Multiple studies hypothesize that intermediate forms of cytochrome c (not fully oxidized, not fully reduced) are responsible for photo-acceptor properties (Karu, 1999, 2010; Karu et al., 2005) and cytochrome c oxidase has recently been confirmed as a photo-acceptor (Passarella and Karu, 2014). Nevertheless, four potential primary light action mechanisms are discussed, which are likely to occur as combinations: (1) alteration of redox properties and acceleration of electrons, (2) changed biochemical activity by local transient heating of chromophores (through conversion of excitation energy into heat energy), (3) production of reactive oxygen species (mainly O_2_^–^) and subsequent H_2_O_2_ production, and (4) photodynamic action and singlet oxygen production (Karu, 1999).

Biological responses of cells after irradiation might play a role as secondary mechanisms (Karu, 1999). The transduction of light action from mitochondria to the nucleus leading to DNA synthesis needs to be elucidated (Karu, 1999). A possible explanation is based on the fact that redox chains (including respiratory chain) are capable of controlling cellular homeostasis. Changes in the redox potential in mitochondria also affect the redox state in the cytoplasm and induce the signal transduction to the nucleus (Karu, 1999). Furthermore, the physiological significance of photosensitivity in enzymes of the respiratory chain is an important question to assess (Karu et al., 2005). Menezes et al. (1998) suggested that IR, for example during sunrise, is a natural process protecting cells from subsequent UV-radiation throughout the day, which is in accordance with conclusions by Applegate et al. (2000) and Burri et al. (2004). Multiple studies have confirmed that IR protects cells from UV cytotoxicity (Danno et al., 1992; Menezes et al., 1998; Frank et al., 2004, 2006). Wavelength-specific influence on cytochrome c oxidase has been reported by Karu et al. (2001; 2003; 2004; 2005) in multiple studies (Karu, 1999, 2008; Passarella and Karu, 2014). Heselich et al. (2012) demonstrated increased genomic instability, increased ROS formation, higher amounts of X-irradiated cells entering the S-phase, and impaired DNA double-strand break repair upon near-infrared-irradiation followed by clinically relevant X-ray doses. These synergistic effects are likely to affect the results of radiotherapy in cancer treatment (Heselich et al., 2012), which is further confirmed by the above-mentioned combination of radiotherapy and heat-therapy with irradiation using wIRA/VIS irradiators (Notter et al., 2017; Vaupel et al., 2018).

Multiple authors have reported increased concentrations of reactive oxygen species (ROS) or decreased concentrations of antioxidative substances upon IR in human skin models *in vitro* or *in vivo* (Schroeder et al., 2007, 2008; Darvin et al., 2009, 2011) and have suspected harmful effects of IR and wIRA on cell layers. Other authors investigated the secretion of matrix-metalloproteinases (MMP) (reviewed in Piazena and Kelleher, 2010). An *in vivo* study investigating IR radiation on albino guinea pigs revealed harmful effects of IR similar to UV-damage (Kligman, 1982). Piazena and Kelleher (2010) give an overview of literature concerning the safety aspects of water-filtered infrared-A or other sources of IR, which the reader is referred to (Piazena and Kelleher, 2010). Most of the studies performed are considered unsuitable as models for infrared or water-filtered IR on the human skin, since most of them lack important information about dose, exposure-time, spectrum, and application of irradiation. Corresponding controls need to be characterized and would help to distinguish the secondary effects of VIS or UV radiation from IR/wIRA effects (Piazena and Kelleher, 2010). Other authors investigating ROS formation in fibroblasts were able to demonstrate increased ROS concentrations upon temperature increase in the samples alone, which leads to the conclusion of ROS activation being an unspecific thermal effect of the radiation (Jung et al., 2010). Therefore, the importance of suitable temperature control needs to be particularly emphasized and is vital in the interpretation of results for *in vitro* and *in vivo* studies (Piazena and Kelleher, 2010; Jung and Grune, 2012).

Even though *in vitro* studies using high doses of wIRA or wIRA/VIS did not induce cell death (Rahn et al., 2016), discomfort in patient due to heating of irradiated tissues might be possible. This can be prevented by either using lower doses [e.g., 1500 – 2000 W/m2 as in Notter et al. (2017)], by keeping a sufficient distance to the irradiator [see Hartel et al. (2006); Künzli et al. (2013) – Table 1, Aljasir et al. (2018)] or by limiting the irradiation duration.

**TABLE 1:** Summary of studies using wIRA as an antibacterialtreatment method.

**Study design**	**Irradiation**	**Experimental setting**	**Specifications**	**Results – bacterial reduction**	**References**
*In vitro/in situ^a^*	wIRA/VIS^1^, BTE31 filter: - Spectrum: 570–1400 nm - Dose: 200 mW/cm^2^ - Duration: 1 min	Irradiation of planktonic cultures (*Streptococcus mutans*, *Enterococcus faecalis*)	aPDT^2^ using toluidine blue (TB^3^) concentrations of 5, 10, 25, and 50 μg/ml carried out in oral cavity of volunteers (*in situ*)	Up to 2 log_10_ reduction of *S. mutans* and *E. faecalis*	Al-Ahmad et al.,2013
		Irradiation of salivary bacteria		Up to 3.7–5 log_10_ reduction of salivary bacteria	
		Irradiation of bacterially colonized bovine enamel slabs		Killing of oral bacterial colonization at all TB concentrations	
*In vitro/in situ^a^*	wIRA/VIS^1^, see Al-Ahmad et al.,2013 - Duration: 5 min	Reduction of CFU^4^ in initial and mature biofilms	aPDT^2^ using TB^3^ or chlorine e6 (Ce6^5^) at a concentration of 100 μg/ml	Initial biofilms: reduction of 3.8 log_10_ Mature biofilms: reduction of 5.7 log_10_	Karygianni et al.,2014
*In vitro/in situ^a^*	wIRA/VIS^1^, see Karygianni et al.,2014 - Distance: 20 cm	Changes of microbial composition in initial and mature biofilms	aPDT^2^ with TB^3^ and Ce6^5^	Reduction up to 3.76 log_10_	Al-Ahmad et al.,2015
*In vitro/ex vivo^b^*	wIRA/VIS^1^, see Karygianni et al.,2014	Irradation of eight different periodontal pathogens	aPDT with Ce6^5^ at a concentration of 100 μg/ml	Reduction of 3.43 – 8.34 log_10_	Al-Ahmad et al.,2016
		Irradation of subgingival biofilms		Reduction of 3.91 – 4.28 log_10_	
Clinical trial	wIRA (590–1400 nm) or VIS (590 - 780 nm): - Duration: 20 min - 2 times/day from day 2 – 10 post-surgery - Distance: 25 cm	Post-surgery irradiation with wIRA or VIS (control) Assessment of wound healing, pain, oxygen partial pressure, and temperature	Double-blinded study	Trend toward a lower rate of wound infections in wIRA group	Hartel et al.,2006
Clinical trial	wIRA (590 – 1400 nm) or VIS (590 – 780 nm): - 20 min pre-operative - Dose: 200 – 225 mW/cm^2^ - Distance: 27 cm	Pre-operative care plus wIRA or VIS (control) irradiation for 20 min, followed by laparotomy Assessment of wound healing, pain and surgical site infections	Double-blinded study Assessment of surgical site infections (SSI) at day 30 post-surgery	Relative risk for SSI in wIRA-treated patients: 0.42 (*p* = 0.018), wIRA acts protective against SSI	Künzli et al.,2013	
Case report	wIRA (Spectrum: N/A^6^) - 30 min - 3 times/day - Distance: 60 cm	Patient with donor site infection after split thickness skin graft – wound cultures: *S. aureus, E. coli, S. epidermidis* (MRSE^7^)	Surgical debridement, removing of hypertrophic granulation tissue, daily wound dressing, and irradiation	Improvement of wound healing: completed or nearly completed wound closure	Aljasir et al.,2018	

*^1^wIRA/VIS, water-filtered infrared-A and visible light irradiation. ^2^aPDT, antimicrobial photodynamic therapy. ^3^TB, toluidine blue. ^4^CFU, colony forming units. ^5^Ce6, chlorine e6. ^6^N/A, not available (no data in publication). ^7^MRSE, methicillin-resistent *Staphylococcus epidermidis.*^a^For detailed material and method section, see Al-Ahmad et al., 2013, 2015 and Karygianni et al., 2014 briefly: *in vitro* examination of bacterial colonies combined with: - Bovine enamel slabs (BES), fixed on jaw splints, transferred into oral cavity of volunteers for 2 h *in situ* (Al-Ahmad et al., 2013). - BES, fixed on upper jaw splints, transferred into oral cavity of volunteers for 2 h or 3 days *in situ* (Karygianni et al., 2014). - BES, see above *in situ* (Al-Ahmad et al., 2015). ^b^For detailed material and method section, see Al-Ahmad et al., 2016 briefly: *in vitro* examination of periodontal pathogens and *ex vivo* subgingival biofilm samples from 6 patients with periodontitis.*

## wIRA as an Antibacterial Treatment Method

A summary of studies using wIRA as an antibacterial treatment method is presented in Table 1. Al-Ahmad et al. (2013) were the first authors to investigate the efficacy of visible light in combination with water-filtered infrared-A (VIS and wIRA) in the killing of bacteria. In their study, antimicrobial photodynamic therapy (aPDT) was tested using toluidine blue (TB) at different concentrations as a photosensitizer and visible light and water-filtered infrared-A radiation as light sources. Bacterial samples tested were planktonic cultures of *Streptococcus mutans* and *Enterococcus faecalis*, bacteria occurring in human saliva or originating from bovine enamel slabs. These investigations were carried out in the oral cavity of healthy volunteers to investigate the effects of aPDT on bacterial colonization *in situ*. All the aforementioned bacteria were severely diminished in load after aPDT treatment, with a maximal reduction of 3.7–5 log_10_ in salivary bacteria (Al-Ahmad et al., 2013). A further study also investigated the action of aPDT consisting of chlorine e6 and wIRA/VIS on multiple periodontal pathogens (*Aggregatibacter actinomycetemcomitans, Porphyromonas gingivalis, Eikenella corrodens, Actinomyces odontolyticus, Fusobacterium nucleatum, Parvimonas micra, Slackia exigua*, and *Atopobium rimae*) and subgingival biofilms *in situ*, which are protected against antibiotic treatment or disinfection by various mechanisms (Al-Ahmad et al., 2016). Karygianni et al. (2014) investigated the effect of aPDT (using toluidine blue or chlorine e6) with wIRA/VIS on initial and mature oral biofilms and observed bacterial reductions of 3.8 and 5.7 log_10_, respectively. *In situ* oral biofilms were severely reduced and the proportion of surviving bacteria was altered compared to controls upon wIRA/VIS radiation, which further encourages the clinical use of wIRA/VIS as treatment for periimplantitis, and periodontitis (Al-Ahmad et al., 2015). Common light sources in aPDT include light-emitting diodes (LED) and wide-band halogen lamps (Al-Ahmad et al., 2016). wIRA/VIS was proven to be beneficial for aPDT therapy in multiple studies (Al-Ahmad et al., 2013, 2016; Karygianni et al., 2014), also including the advantages of using a broader wavelength-spectrum than LED, reduced risk of tissue overheating compared to halogen lamps, and its well-known beneficial effects on wound healing (Al-Ahmad et al., 2016).

In addition to the above-mentioned beneficial aspects of wIRA/VIS in aPDT, the application of wIRA/VIS alone (without photosensitizer) had beneficial effects on wound infections after abdominal surgery (Hartel et al., 2006; Künzli et al., 2013). The case report of a patient with a donor site infection in a split-thickness skin graft is especially worth mentioning, since *Staphylococcus aureus, Escherichia coli*, and *Staphylococcus epidermidis* (Methicillin-resistant, MRSE) were isolated from the wound site and the combination of surgical intervention, daily wound dressing, and combined wIRA/VIS treatment led to almost complete healing of the infected sites within 5 weeks (Aljasir et al., 2018).

Finally, not only extracellular bacteria, wound infections and biofilm formation can be treated with wIRA/VIS, but this irradiation technique has also been demonstrated to reduce acute chlamydial infections *in vitro* (Marti et al., 2014, 2015; Rahn et al., 2016; Kuratli et al., 2018). The reducing effects of wIRA/VIS on chlamydial infectivity were confirmed in multiple *in vitro* studies, using animal (Vero cells and *C. pecorum*) and human cell culture models (a genital model with HeLa cells and *C. trachomatis* Serovar E or an ocular model with human conjunctival epithelial cells and *C. trachomatis* Serovar B), different chlamydial infectious doses, different irradiation time points, irradiation doses or wavelengths (wIRA alone (black filter, 780–1400 nm), wIRA/VIS (orange filter, 595–1400 nm or no filter, 380–1400 nm), and temperature controlled settings (Marti et al., 2014, 2015; Rahn et al., 2016; Kuratli et al., 2018). Results of previous studies investigating wIRA or wIRA/VIS for the treatment of chlamydial infections are summarized in Table 2.

**TABLE 2:** Summary of studies investigating the effects of wIRA or wIRA/VIS in different cell-*Chlamydia* systems.

		**Cell + *Chlamydia* model^3^**	**Time-point of irradiation^4^**	**Duration + dose [W/m^2^]^5^**	**Results^6^**	**References**
wIRA^1^	Single dose	HCjE (prim), HeLa (perm), Vero (perm), CtB, CtE, and Cp	before infection, 0 hpi, and 40 hpi	20/30 min, 650 or 2000	IFA: Inclusion rate ↓	Marti et al., 2015; Rahn et al.,2016
					Titer: infectivity ↓ (2000),	
					infectivity ↓ (650: only Cp + Vero)	
			→EB irradiation: infectivity reduction dependent on VIS			
	Quadruple dose	HCjE (prim) + CtB	Before infection (8, 5, 3, and 0 h)	30 min, 2000	Titer: infectivity ↓	Rahn et al.,2016
wIRA/VIS^2^	Single dose	HCjE (prim), HeLa (perm), Vero (perm), CtB, CtE, and Cp	Before infection, 0 hpi, 40 hpi	20/30 min, 1000, 2000, 2100, or 3700	IFA: inclusion rate ↓	Marti et al., 2014; Marti et al., 2015; Rahn et al.,2016
					Titer: infectivity ↓	
					TEM: number of chlamydial	
					particles in inclusion ↓	
					TcS: Non-thermal effects involved	
	Triple dose	HeLa + CtE	24, 36, and 40 hpi	20 min, 3700	IFA: inclusion rate ↓	Marti et al.,2014
					Titer: infectivity ↓	
					TEM: number of chlamydial	
					particles in inclusion ↓, EBs ↓,	
					RBs ↑	
					Array: cytokine/chemokine ↑	
	Triple dose	HeLa + CtE	24, 36, and 40 hpi	30 min, 2340 – 3400	IFA: inclusion size ↓	Kuratli et al.,2018
					Titer: infectivity ↓	
					ELISA: IL-6 ↑, IL-8 ↑, RANTES ↑	
	Quadruple dose	HCjE (prim) + CtB	Before infection (8, 5, 3, and 0 h)	30 min, 2100	Titer : infectivity ↓	Rahn et al.,2016

*^1^wIRA, water-filtered infrared-A (wavelengths 780–1400 nm). ^2^wIRA/VIS, water-filtered infrared-A and visible light [380 – 1400 nm or 595 – 1400 nm (Rahn et al., 2016)]. ^3^Prim, primary cell line; Perm, permanent cell line; HCjE, human conjunctival epithelial cells; HeLa, human cervical carcinoma cells; Vero, African green monkey kidney cells; CtB, *Chlamydia trachomatis* serovar B (ocular strain); CtE, *Chlamydia trachomatis* serovar E (genital strain); Cp, *Chlamydia pecorum.*^4^Before infection, only irradiation of cells. 0 hpi, hours post infection (hpi). Irradiation of elementary bodies (EBs) directly before infection. 40 hpi, 40 h post infection. ^5^Duration, duration of irradiation exposure; W/m^2^, irradiation intensity/dose (in Watts per square meter). ^6^IFA, immunofluorescence assay – determination of inclusion frequency, morphology, or size. Titer, titration by subpassage to analyze chlamydial infectivity (IFU/ml, inclusion forming units per ml); TEM, transmission electron microscopy – information about ultrastructure of chlamydial inclusions; TcS, temperature-controlled setting (water bath set to 34°C vs. 37°C).*

**FIGURE 1:**
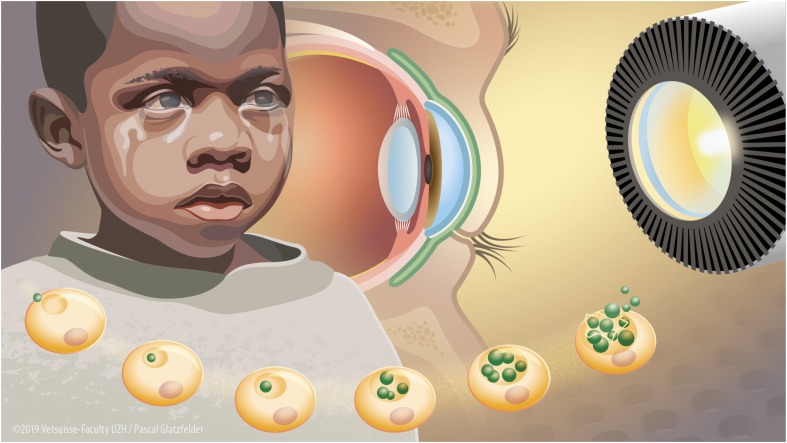
Water-filtered infrared-A and visible light irradiation (wIRA/VIS) as a potential future treatment device to combat blinding trachoma. Blinding trachoma is a neglected tropical disease present in Sub-Saharan Africa, in particular affecting children. It is caused by recurrent infections of the conjunctival epithelium by *C. trachomatis* leading to fibrosis and scarring of the conjunctiva, ocular discharge, trichiasis, and corneal opacity with blindness (left aspect of the figure). *C. trachomatis* is an obligate intracellular bacterium with a complex life cycle including the attachment of an elementary body (EB) to an epithelial cell, forming an intracytoplasmic inclusion with differentiation into reticulate bodies and multiplication by binary fission and finally lysis of the inclusion with the release of EBs (bottom aspect of the figure). wIRA/VIS irradiation produced by radiation of a halogen bulb, filtered through a water-filled cuvette in special irradiators (right aspect of the figure) could reduce chlamydial burden *in vitro* by affecting mature inclusions and as well as by reducing infectious EB load. Future *in vivo* wIRA/VIS treatment of trachoma patients would be applied by irradiation of the *C. trachomatis*-infected conjunctiva (in green) through a closed eyelid reaching the infected part (inner conjunctival lining) without harming the inner structures of the eye such as the cornea, the vitreous body or the retina (central aspect of the figure).

Since chlamydiae are obligate intracellular bacteria, the first investigations of wIRA treatment for chlamydial infections focused on possible deteriorating effects of wIRA irradiation on host cells. Detrimental effects on host cells were not present in HeLa (human cervical carcinoma cells) and Vero (African green monkey kidney) cells (Marti et al., 2014). In primary human conjunctival epithelial cells (HCjE), wIRA treatment reduced cell metabolism as measured by Alamarblue assay, whereas wIRA/VIS did not. However, the additional counting of cell numbers per high power field (400× magnification) did not show decreased cell numbers after either wIRA/VIS or wIRA irradiation treatment (Rahn et al., 2016). Following these findings, further investigations included *ex vivo* models, which are important for potential future application of wIRA as a trachoma treatment method (Figure 1). Although a temperature increase was demonstrated in constantly perfused pig eyes upon high irradiation doses, no wIRA/VIS-dependent phosphorylation of stress kinases was observed in retinal explants from adult or postnatal (10-day-old) mice (Rahn et al., 2016).

Irradiation of HCjE cells alone prior to chlamydial infection reduced subsequent chlamydial infectivity, indicating a cell-associated, protective effect of wIRA/VIS against chlamydial infections (Rahn et al., 2016). Irradiating elementary bodies (EBs – infective particles in the chlamydial life cycle) prior to infection of host cells also reduced chlamydial infectivity (Marti et al., 2014, 2015; Rahn et al., 2016) and the combination of both prior to infection further increased this effect (Rahn et al., 2016).

The irradiation of mature chlamydial inclusions *in vitro* with either wIRA or wIRA/VIS at doses of 2000 or 3700 W/m^2^ led to a decrease of the number of infective particles in the chlamydial inclusions without causing changes in the distribution of chlamydial maturation stages (Marti et al., 2014, 2015). Triple dose irradiation of growing *Chlamydia*, on the other hand, led to increased proportions of reticulate bodies (RBs – the reproducing form of *Chlamydi*a) and decreased EB formation, whereas no increased formation of aberrant bodies [AB – a sign of the chlamydial stress response (Bavoil, 2014)] could be observed (Marti et al., 2014). The distribution differences in chlamydial development stages indicate a yet unknown effect of wIRA/VIS on the chlamydial development cycle (Marti et al., 2014).

## Future Research Directions

To elucidate the mechanism of wIRA/VIS in antibacterial effects or in wound healing, transcriptomics or metabolomics would provide useful information regarding gene expression and protein translation upon irradiation. Since cytochrome c oxidase has been claimed to be the primary photo-acceptor molecule, investigating the molecule itself, and the broader mitochondrial metabolism might provide useful information regarding biological effects following wIRA irradiation.

Various bacterial species have been demonstrated to display thermo-sensitivity, therefore local thermal therapies (as e.g., wIRA therapy) are important fields for future investigations (Gazel and Yılmaz, 2018). Heat treatment has further been proven beneficial for *Leishmania*-induced skin lesions (Aronson et al., 2010) and buruli ulcer, another tropical disease, which is caused by *Mycobacterium ulcerans* (Junghanss et al., 2009; Vogel et al., 2016). These neglected tropical diseases might represent further possible indications for the use of wIRA.

In view of current worldwide antibiotic resistance problems, studies investigating wound infection treatment with heat and light have become more important in the last few decades, with promising results for these treatments. Further investigations are needed to gain deeper insight into the working mechanisms of such methods, their safety aspects, and applicability in patients.

## Author Contributions

JK and NB drafted the manuscript.

## Conflict of Interest Statement

The authors declare that the research was conducted in the absence of any commercial or financial relationships that could be construed as a potential conflict of interest.
